# Depressive symptoms, violence, and discrimination among transgender women during the COVID-19 pandemic: a cross-sectional study

**DOI:** 10.17843/rpmesp.2025.423.14287

**Published:** 2025-09-12

**Authors:** Renato A. Errea, Noris G. Hernandez, Janeth Santa-Cruz, Carmen Contreras, Lourdes Ramos, Diego Rondón, Karen Ramos, Leyla Huerta, Leonid Lecca, Jesús Peinado

**Affiliations:** 1 Socios En Salud Sucursal Perú, Lima, Peru. Socios En Salud Sucursal Perú Lima Peru; 2 School of Social Work, University of South Florida, Tampa, FL, USA. University of South Florida School of Social Work University of South Florida Tampa FL USA; 3 Escuela Profesional de Tecnología Médica, Universidad Privada San Juan Bautista, Lima, Peru. Universidad Privada San Juan Bautista Escuela Profesional de Tecnología Médica Universidad Privada San Juan Bautista Lima Peru; 4 Asociación Féminas Perú, Lima, Peru. Asociación Féminas Perú Lima Peru; 5 Departamento de Salud Global y Medicina Social, Escuela de Medicina de Harvard, Boston, Estado Unidos. Departamento de Salud Global y Medicina Social Escuela de Medicina de Harvard Boston Estado Unidos; 6 Escuela de Medicina, Facultad de Ciencias de la Salud, Universidad Peruana de Ciencias Aplicadas, Lima, Peru. Universidad Peruana de Ciencias Aplicadas Escuela de Medicina Facultad de Ciencias de la Salud Universidad Peruana de Ciencias Aplicadas Lima Peru

**Keywords:** Transfeminine Persons, Mental Health, Depression, Gender Discrimination, Gender-Based Violence, Peru

## Abstract

**Objective.:**

To examine the frequency of depressive symptoms, gender-based violence, and gender-based discrimination among transgender women (TGW) in Lima, Peru, during the COVID-19 pandemic.

**Materials and Methods.:**

Between May and July 2021, the non-governmental organization Socios En Salud conducted a cross-sectional study to assess depressive symptoms, gender-based discrimination and violence among TGW identified from a prior study database and peer referral. Descriptive statistics were used to report frequencies, and an exploratory bivariate analysis was performed to examine associations between depressive symptoms and participants´ characteristics, including experiences of discrimination and violence. Depressive symptoms were assessed using the Patient Health Questionnaire-9; gender-based discrimination and violence were measured using adopted instruments.

**Results.:**

A total of 112 participants were included in the study. Most TGW reported depressive symptoms (81.2%), experiences of gender-based violence (83.8%), and experiences of gender-based discrimination (95.5%). The presence of depressive symptoms was associated with experiences of discrimination in healthcare settings (*p* < 0.046) and with experiences of violence (*p* < 0.046).

**Conclusions.:**

A high frequency of depressive symptoms, as well as experiences of gender-based discrimination and violence, was observed among TGW in Lima, Peru, during the COVID-19 pandemic. In addition, significant associations were identified between these experiences and the presence of depressive symptoms. These findings are consistent with existing evidence on the mental health impact of the pandemic, but also suggest a disproportionate burden among TGW and highlight the urgent need to address the underlying contributing factors.

## INTRODUCTION

Transgender women (TGW) have a higher prevalence of depression compared to the general population. For example, in the United States, studies estimated a lifetime prevalence of depression in TGW of 62%, while in the general population it was estimated at 17% ^1^^,^^2^. Likewise, compared to other sexual minorities, such as gay and bisexual men, TGW more frequently report depressive symptoms ^3^^,^^4^. Depression impairs overall health and is the main risk factor for suicide ^5^^,^^6^. Consistent with this, evidence also shows higher rates of suicidal behaviors in transgender people compared to the general population and other gender-diverse groups ^7^^,^^8^.

Several psychosocial factors contribute to the high prevalence of depression in TGW. Among them, stigma, discrimination, and different types of violence (psychological, physical, and sexual) have been linked to the development of depressive disorders in this population ^4^^,^^9^^-^^13^. Many TGW live immersed in a cycle of discrimination and violence that often begins in childhood or adolescence, driven by the lack of acceptance of non-normative gender identities by their families and society ^14^. Accordingly, rates of discrimination and violence against TGW are high in different countries around the world with different cultures ^15^^-^^17^.

These experiences of discrimination and violence promote social exclusion and limit access to educational opportunities and formal employment ^14^. In Peru, for example, about 64% of TGW have not completed primary education and approximately 72% engage in sex work as a means of subsistence ^18^. This situation favors the acquisition of health conditions associated with both risk behaviors and poverty. For example, in the country, approximately 30% of TGW live with HIV and about 3.5% have active tuberculosis ^19^^,^^20^.

On the other hand, the COVID-19 pandemic generated multiple stressors such as insecurity, fear, death of close ones, socioeconomic losses, and significant changes in daily life, which considerably affected the mental health of the population ^21^. A systematic review estimated that the global prevalence of depression increased during the pandemic, exacerbating the already high burden of this disease before the pandemic context ^22^. In TGW, studies conducted during the pandemic also reported a high frequency of severe psychological distress symptoms, with high levels of depressive symptomatology, higher than those observed in other sex-gender minorities ^23^^,^^24^.

Peru was one of the countries most affected by the COVID-19 pandemic, with the highest reported mortality worldwide ^25^. Despite this, there is scarce evidence on its effect on the frequency of depressive symptoms in a highly vulnerable population such as the TGW community. To contribute to closing this knowledge gap, the objective of this study was to describe the presence of depressive symptoms, gender-based discrimination and violence, as well as to explore the possible factors associated with depressive symptomatology in TGW in Lima, Peru, during the COVID-19 pandemic.

**KEY MESSAGES**

Motivation for the study. Transgender women (TGW) are a socially and historically stigmatized and marginalized population with high rates of mental health disorders, including depression. The COVID-19 pandemic generated multiple stressors that deteriorated people’s mental health.

Main findings. The findings of this study showed a disproportionately high frequency of depressive symptoms in TGW in Lima, Peru, compared to reports in the general population. Likewise, high levels of gender-based discrimination and violence were observed, consistent with what has been reported in other countries.

Implications. These results highlight the importance of structurally addressing stigma, discrimination, and violence against TGW, including the effective implementation of anti-discrimination policies and laws.

## MATERIALS AND METHODS

### Study type and design

The non-governmental organization Socios En Salud Sucursal Perú (SES) conducted a cross-sectional study with the objectives of determining the frequencies of mental health problems, tuberculosis (TB), and TB/HIV coinfection in TGW residing in Lima. Questionnaires were used to identify possible mental health problems, chest radiography and molecular tests were used for TB detection, and rapid tests or self-report information were used to determine HIV serostatus. TGW identified with any of these conditions received support to receive the respective treatment from SES professionals or in public health facilities. In this article, the results of the mental health component are reported, describing the frequencies of depressive symptoms, gender-based discrimination, and gender-based violence in the participants. The results of the TB and TB/HIV coinfection component have been previously reported ^20^.

### Population characteristics and sample selection

Potential study participants were 18 years or older, resided in Metropolitan Lima, were approached between May and July 2021, and were identified by non-probabilistic sampling using two sources: a) the database of participants from a cross-sectional study conducted by SES in 2020 whose objective was to describe access to medical care services in TGW residing in Lima, or b) direct peer referral ^26^. TGW who were contacted and agreed to participate in the study provided their consent to be evaluated for one or more of the three study components (mental health, TB, or TB/HIV coinfection). The participants included in this article were those who agreed to be evaluated for the mental health component.

During the participant recruitment period, Peru was in its second wave of COVID-19. By July 2021, Peru had reported more than 2 million cases and nearly 2,500 deaths from COVID-19; just under half of the cases and a quarter of the deaths came from Lima ^27^.

### Measurement and categorization of variables

The main variable of interest in the study was the presence of depressive symptoms. To identify these symptoms, participants evaluated for mental health disorders had to first complete the Patient Health Questionnaire-2 (PHQ-2). The PHQ-2 is a two-question tool that measures the frequency of anhedonia and depressed mood during the last two weeks ^28^. Participants who tested positive on either of the two PHQ-2 questions had to complete the Patient Health Questionnaire-9 (PHQ-9). The PHQ-9 questionnaire is a nine-question tool that assesses depressive symptoms more comprehensively and evaluates their severity according to the following scoring system: no depressive symptoms (score: 0-4), with mild depressive symptoms (score: 5-9), with moderate depressive symptoms (score: 10-14), with moderately severe depressive symptoms (score: 15-19), and with severe depressive symptoms (score: ≥20) ^(^^29^^,^^30^. The Spanish version of the PHQ-9 used in the study has received expert validation in Peru; likewise, a local study showed adequate levels of reliability and validity for a unidimensional model of said questionnaire ^31^^,^^32^.

The secondary variables of interest were gender-based discrimination and violence. For this, the content of a questionnaire created and used by the Latin American Center for Sexuality and Human Rights for a survey of members of the LGTBIQ+ community in Bogotá, Colombia, in 2007 was adopted, with slight modifications ^33^. From this questionnaire, questions referring to discrimination and violence were identified to collect variables referring to experiences of gender-based discrimination and the environment where this discrimination was experienced, as well as experiences of gender-based violence, the type of violence experienced, and the environment where this violence was experienced. The details of the adopted questions and their operationalization are shown in the supplementary material.

Finally, information was collected on other variables of interest for the study, such as HIV serostatus, either by self-report or by performing rapid HIV tests; as well as sociodemographic information corresponding to the age, marital status, educational level, and occupation of the participants.

### Statistical analysis

A descriptive analysis was performed, reporting the median and interquartile range for age (after determining non-compliance with a normal distribution with the Shapiro-Wilk test), and absolute and relative frequencies for the rest of the categorical variables such as the sociodemographic characteristics of the participants and the variables of depressive symptoms, gender-based discrimination, and gender-based violence. Exploratorily, a bivariate analysis was carried out to identify the association between the presence of depressive symptoms and demographic characteristics, HIV serostatus, gender-based discrimination, and gender-based violence. For the bivariate analysis, the PHQ-9 result was dichotomized into “without depressive symptoms” (PHQ < 5) and “with depressive symptoms” (PHQ ≥ 5); this cut-off point is the one indicated by the Ministry of Health of Peru to classify the result of depression screening with the PHQ-9 questionnaire as “positive” or “negative,” where a “positive” result means a possible diagnosis of depression and indicates, within the care flowchart, the need to receive a mental health consultation for diagnostic confirmation and treatment, if applicable ^34^. For this bivariate analysis, the Mann-Whitney U test was used for comparison with age, Fisher’s exact test for categorical variables when the expected number in more than 20% of the cells was less than five, and the chi-square test for categorical variables that did not meet the criteria for using Fisher’s exact test. Statistical significance was considered for p-values less than 0.05. Participants with missing data were excluded from the analysis. Data analysis was performed using the statistical program STATA v17.0 (Stata Corporation, College Station, Texas, USA).

### Ethical aspects

The protocol of the main study was approved by the Institutional Committee of Ethics in Research of Socios En Salud with code #004, complying with internationally required ethical standards. All participants provided their written informed consent. Data management and analysis were performed with non-identifiable information of the participants.

## RESULTS

**Figure 1:**
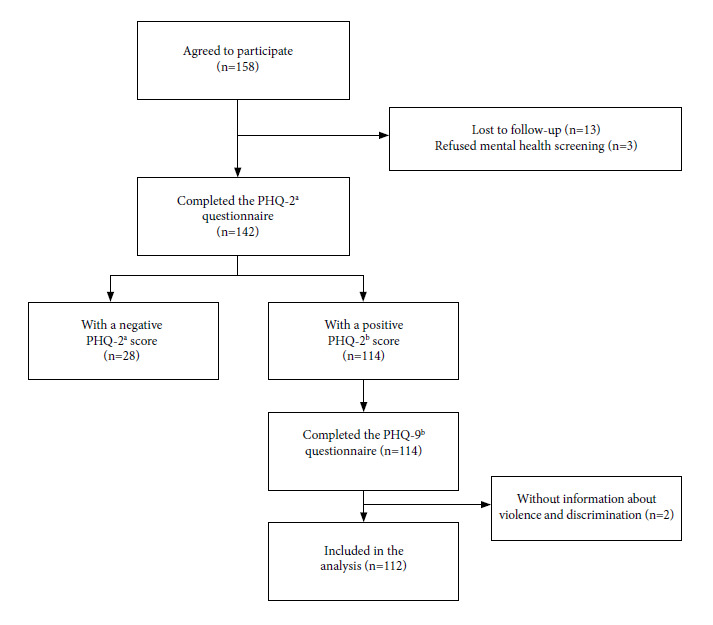
Flowchart of study participants.

^a^ PHQ-2, Patient Health Questionnaire 2; b PHQ-9, Patient Health Questionnaire 9

A total of 158 participants gave their consent to participate in the study. Of these, 142 TGW completed the PHQ-2 questionnaire. According to the PHQ-2 responses, 114 TGW were eligible to complete the PHQ-9 questionnaire. Among the 114 who answered the PHQ-9 questionnaire, two TGW were excluded due to missing data on violence and discrimination, resulting in the inclusion of 112 participants in the analysis (Figure 1).

The median age of the 112 study participants was 33.2 years (IQR: 11.1) and 21.6% had not completed school education. The most frequent occupations were sex work (32.1%) and hairstyling (17.0%); while 33.9% were unemployed. Almost a third (29.5%) were living with HIV (Table 1).

**Table 1:** Characteristics of 112 transgender women in Lima, Peru, 2021.

Characteristic		Total n (%)
Age, median (interquartile range)		33.2 (11.1)
Marital status		
	Single	105 (93.8)
	Cohabiting	7 (6.2)
Educational level		
	Completed primary school or less	4 (3.6)
	Incomplete secondary school	19 (17.0)
	Completed secondary school	51 (45.5)
	Higher education	38 (33.9)
Occupation		
	Sex worker	36 (32.0)
	Stylist	19 (17.0)
	Merchant	5 (4.5)
	Health worker	4 (3.6)
	Cook/flight attendant	3 (2.7)
	Other	7 (6.3)
	Unemployed	38 (33.9)
Living with HIV		
	Yes	33 (29.5)
	No	79 (70.5)
Has ever experienced gender-based discrimination		
	Yes	107 (95.5)
	No	5 (4.5)
Setting where gender-based discrimination was experienced (n=107)		
	Workplace	65 (60.8)
	Family/close environment	83 (77.6)
	Health facilities	50 (46.7)
	Religious facilities	32 (29.9)
	Educational institutions	54 (50.5)
	Judicial/police facilities	84 (78.5)
	Recreational spaces	51 (47.7)
Has ever experienced gender-based violence		
	Yes	94 (83.9)
	No	18 (16.1)
Type of gender-based violence experienced (n=94)		
	Physical	46 (48.9)
	Psychological	94 (100.0)
	Sexual	22 (23.4)
Setting where gender-based violence was experienced (n=94)		
	Family/close environment	16 (17.0)
	Workplace	5 (5.3)
	Educational institutions	4 (4.3)
	Recreational spaces	54 (57.5)
	Health facilities	0 (0.0)
	Not specified	15 (16.0)
Depressive symptoms		
	No symptoms	21 (18.8)
	Mild symptoms	54 (48.2)
	Moderate symptoms	23 (20.5)
	Moderately severe symptoms	11 (9.8)
	Severe symptoms	3 (2.7)

a Median (interquartile range). The “age” variable did not show a normal distribution (Shapiro-Wilk test: W = 0.954, p-value < 0.001)

Regarding experiences of discrimination due to their gender identity, 95.5% reported having suffered discrimination, among them, the most frequent settings were in judicial/police facilities (78.5%) and in the family/close environment (77.6%). 83.9% reported experiences of violence due to their gender identity, with psychological violence being the most frequent (100%), and the most common setting being recreational spaces (57.5%), followed by the family/close environment (17.0%). Most participants had some degree of depressive symptoms (81.2%), with a predominance of mild depressive symptoms (48.2%) and moderate depressive symptoms (20.5%).

The exploratory analysis showed a statistically significant association between those participants who experienced gender-based discrimination in health facilities (p = 0.046) and between those who experienced gender-based violence (p = 0.042) (Table 2).

**Table 2:** Bivariate analysis of depressive symptoms and other characteristics of 112 transgender women in Lima, Peru, 2021.

Characteristic			Without depressive symptoms (N=21) n (%)	With depressive symptoms (N=91) n (%)	p-value
Age, median, (interquartile range)			35.0 (8.8)	32.8 (11.7)	0.751^a^
Marital status					
	Single		21 (20.0)	84 (80.0)	0.344^b^
	Cohabiting		0 (0.0)	7 (100.0)	
Educational level					
	Secondary education or less		12 (16.2)	62 (83.8)	0.338^c^
	Higher education		9 (23.7)	29 (76.3)	
Occupation					
	Sex worker		6 (16.7)	30 (83.3)	0.448^b^
	Stylist		2 (10.5)	17 (89.5)	
	Others		6 (31.6)	13 (68.4)	
	Unemployed		7 (18.4)	31 (81.6)	
Person living with HIV					
	Yes		7 (21.2)	26 (78.8)	0.666^c^
	No		14 (17.7)	65 (82.3)	
Experience of gender-based discrimination					
	Yes		20 (18.7)	87 (81.3)	1.00^b^
	No		1 (20.0)	4 (80.0)	
Setting where gender-based discrimination was experienced (n=107)					
	Workplace				
		Yes	11 (16.9)	54 (83.1)	0.559^c^
		No	9 (21.4)	33 (78.6)	
	Family/close environment				
		Yes	14 (16.9)	69 (83.1)	0.382^b^
		No	6 (25.0)	18 (75.0)	
	Health facilities				
		Yes	5 (10.0)	45 (90.0)	0.031^c^
		No	15 (26.3)	42 (73.7)	
	Religious facilities				
		Yes	5 (15.6)	27 (84.4)	0.595^c^
		No	15 (20.0)	60 (80.0)	
	Educational institutions				
		Yes	8 (14.8)	46 (85.2)	0.299^c^
		No	12 (22.6)	41 (77.4)	
	Judicial/police facilities				
		Yes	16 (19.0)	68 (81.0)	1.000^b^
		No	4 (17.4)	19 (82.6)	
	Recreational spaces				
		Yes	7 (13.7)	44 (86.3)	0.209^c^
		No	13 (23.2)	43 (76.8)	
Experience of gender-based violence					
	Yes		14 (14.9)	80 (85.1)	0.042^c^
	No		7 (38.9)	11 (61.1)	
Type of gender-based violence experienced (n=94)					
	Physical				
		Yes	8 (17.4)	38 (82.6)	0.506^c^
		No	6 (12.5)	42 (87.5)	
	Psychological				
		Yes	14 (14.9)	80 (85.1)	N/A^d^
		No	0 (0.0)	0 (0.0)	
	Sexual				
		Yes	6 (27.3)	16 (72.7)	0.086^b^
		No	8 (11.1)	64 (88.9)	
Setting where gender-based violence was experienced (n=94)					
	Family/close environment				
		Yes	2 (12.5)	14 (87.5)	1.000^b^
		No	12 (15.4)	66 (84.6)	
	Workplace				
		Yes	1 (20.0)	4 (80.0)	0.562^b^
		No	13 (14.6)	76 (85.4)	
	Educational institutions				
		Yes	0 (0.0)	4 (100.0)	1.000^b^
		No	14 (15.6)	76 (84.4)	
	Recreational spaces				
		Yes	6 (11.1)	48 (88.9)	0.231^c^
		No	8 (20.0)	32 (80.0)	

a The p-value was calculated using the Mann-Whitney U test.

b The p-value was calculated using Fisher’s exact test.

c The p-value was calculated using the chi-square test.

d Statistical test not applicable: row with frequency 0.

## DISCUSSION

A high frequency (81.2%) of depressive symptoms was found in TGW in Lima, Peru, during the COVID-19 pandemic. A local study in TGW in Lima conducted before the pandemic reported a lower frequency of depressive symptoms (PHQ ≥ 5) in TGW compared to the present study (60% vs 81.2%, respectively), although that study had a small sample size ^35^. Likewise, the frequency of moderate to more severe depressive symptoms (PHQ ≥ 10) was higher than the prevalence reported by a local study conducted during the pandemic in the general population (33.0% vs 19.7%, respectively) ^36^. These findings suggest that the stressful conditions of the pandemic have disproportionately affected the mental health of TGW compared to the general population, generating high depressive symptomatology.

Violence (83.9%) and gender-based discrimination (95.5%) showed high figures in the study population. Regarding violence, high frequencies of physical (48.9%) and psychological (100%) violence were reported, while sexual violence was lower (23.4%). With the exception of sexual violence, these findings are comparable to those reported in a study conducted in Brazil, which found high prevalences of sexual (47.5%), physical (54.2%), psychological (85.8%) violence, and discrimination (91.6%) ^37^. On the other hand, a study in Iran reported a similar frequency of psychological violence (92%), with a higher frequency of physical (70.9%) and sexual (63%) violence ^15^. Despite some variations, these findings reflect the magnitude of the violence and discrimination faced by transgender women in different sociocultural contexts.

In the present study, the exploratory bivariate analysis identified a significant association between depressive symptoms during the pandemic and gender-based violence. This finding is consistent with the literature that documents positive associations between violence (psychological, physical, or sexual) and major depression in non-pandemic contexts ^9^^,^^10^. Likewise, a significant association was observed between the presence of depressive symptoms and experiences of gender-based discrimination in healthcare settings. This result is particularly important in the Peruvian context, where a study conducted in five regions reported that about 30% of TGW had suffered discrimination in a health facility in the last twelve months ^(^^38^; these same experiences of discrimination can lead to a refusal to seek mental health services in these facilities. The mental health of TGW can be favored through the implementation of inclusive health services ^39^. However, to comprehensively address violence and discrimination, it is essential to promote effective anti-discriminatory legal frameworks ^40^, and to develop interventions aimed at the perpetrators themselves ^41^.

The frequency of depressive symptoms between TGW living with HIV and those who were not was similar (78.8% and 82.8%, respectively). Consistently, a cross-sectional study in Brazil that used the PHQ-9 questionnaire also found no differences in the presence of depressive symptoms according to HIV serostatus in TGW ^9^. However, the literature indicates that depression is a predisposing factor for engaging in risky sexual practices in TGW ^42^, even higher than when compared to men who have sex with men ^43^. Considering that one of the main points of contact of TGW with the health system are HIV and sexually transmitted infection (STI) services, either for screening tests or for access to treatment, a key opportunity is presented to integrate mental health care within these services ^44^. This integration becomes more relevant given the shortage of specialized mental health personnel in many low- and middle-income countries, such as Peru. In relation to this, low-intensity interventions for the management of depression (i.e., interventions implemented by non-specialized but trained mental health personnel) have proven to be effective in HIV/STI services in these contexts ^45^. Implementing these strategies in the country could facilitate access to mental health services for TGW.

In pandemic contexts, characterized by severe mobility restrictions such as quarantines, digital health-based interventions can reduce psychological stress and depressive symptomatology. In line with this, a meta-analysis revealed that interventions based on cognitive-behavioral therapy delivered through telemedicine were effective in reducing symptoms of depression in the general population during the COVID-19 pandemic ^46^. These interventions varied in their format: some were conducted by health personnel, others were self-guided; some were offered in abbreviated formats and others in more extensive formats. In contrast, evidence on the use of digital mental health in sexual and gender minorities during this period is scarce. A quasi-experimental study implemented during the COVID-19 pandemic in a sample of people belonging to sexual and gender minorities, including transgender people, showed that a brief, three-session intervention delivered by peers, managed to significantly reduce depressive symptoms ^47^. These findings demonstrate the potential of digital mental health interventions during pandemic scenarios, but also highlight the urgent need to generate specific and adapted evidence for sexual and gender minorities.

This study has limitations. First, participants who scored negative on the PHQ-2 questionnaire were excluded from the analysis, which could have overestimated the percentage of people with depressive symptoms; this exclusion was because, in these participants, data on the other relevant variables of the study were not collected. Second, although depressive symptoms were measured using the PHQ-9 questionnaire, a validated tool in Peru and widely recommended for depression screening in different contexts ^31^^,^^32^^,^^48^, it has not been validated or adapted for use in the transgender population in Peru, which could have affected the reliability and validity of the results. Similarly, the instruments used to collect information on gender-based discrimination and violence were not validated or adapted to the context of the transgender population; which could also have influenced the reliability and validity of these measurements. On the other hand, the non-probabilistic sampling used could have introduced selection biases. In addition, peer-referral sampling could have introduced additional selection biases by favoring the inclusion of participants with similar social or behavioral characteristics. Both factors limit the external validity of the results. Finally, the limited number of participants restricted the statistical power of the study and did not allow for adjusted analyses. However, it is important to consider that TGW are a hard-to-reach population and that other studies in this population face similar sampling challenges.

In conclusion, a high frequency of depressive symptoms, gender-based discrimination, and violence was evidenced in TGW in Lima, Peru, during the COVID-19 pandemic; as well as associations of experiences of discrimination and violence with the presence of depressive symptomatology. The findings suggest a disproportionate impact of the COVID-19 pandemic on the mental health of TGW, which underscores the importance of addressing the structural and social factors that generate it.
