# Supplementary material for: Depressive symptoms, violence, and discrimination among transgender women during the COVID-19 pandemic: a cross-sectional study
Source: Rev Peru Med Exp Salud Publica. 2025 Sep 12;42(3):281–90. doi: 10.17843/rpmesp.2025.423.14287 (PMC12679979; doi:10.17843/rpmesp.2025.423.14287)
Supplement: Supplementary material. — Available in the electronic version of the RPMESP. [file rpmesp-42-03-14287-s001.docx]

**Material suplementario**

**Cuestionario de discriminación y violencia**

**Enunciado:** Ahora voy a hacerle unas preguntas sobre las situaciones difíciles que usted ha experimentado a causa de su identidad sexual. Yo entiendo que puede ser incómodo hablar de estas cosas pero por favor recuerde que lo que usted me diga es completamente confidencial y que sus respuestas nos ayudarán a entender mejor las necesidades de la población LGBT en nuestra sociedad.

**Pregunta 1. A causa de su identidad sexual ¿Alguna vez ha sufrido alguna de las siguientes discriminaciones? (Opción múltiple)**

No ha sido empleada o contratada o ha sido despedida de su trabajo 🡪 *Operacionalización: Discriminación en entorno laboral*

Le prohibieron la entrada o permanencia en algun comercio o de entretenimiento 🡪 *Operacionalización: Discriminación en espacios recreativos/públicos*

Ha sido mal atendida en servicios de salud por profesionales de la salud 🡪 *Operacionalización: Discriminación en establecimientos de salud*

Ha sido discriminada por profesores(as) o compañeros(as) en colegio/ instituto o universidad 🡪 *Operacionalización: Discriminación en instituciones educativas*

Ha sido discriminada por amigos(as) 🡪 *Operacionalización: Discriminación en entorno cercano/familiar*

Ha sido discriminada por vecinos(as) 🡪 *Operacionalización: Discriminación en entorno cercano/familiar*

Ha sido excluida o marginada del ambiente familiar 🡪 *Operacionalización: Discriminación en entorno cercano/familiar*

Ha sido excluida o marginada de un espacio o grupo religioso 🡪 *Operacionalización: Discriminación en instalaciones religiosas*

Se le ha impedido donar sangre 🡪 *Operacionalización: Discriminación en establecimientos de salud*

Ha sido discriminada por policias/ serenazgo 🡪 *Operacionalización: Discriminación en instalaciones judiciales/policiales*

Ha sido discriminada por personal de seguridad privada 🡪 *Operacionalización: Discriminación en instalaciones judiciales/policiales*

Ha sido mal atendida en comisaria o juzgados de familia 🡪 *Operacionalización: Discriminación en instalaciones judiciales/policiales*

Le han negado un derecho en una instancia judicial (comisarias / juzgados) 🡪 *Operacionalización: Discriminación en instalaciones judiciales/policiales*

Ha sufrido otra forma de discriminación, ¿cual? _______ 🡪 *Operacionalización:* *(de acuerdo a respuesta brindada)*

Nota:

Si marcó al menos uno casilla en pregunta 1 🡪 *Operacionalización: Sí discriminación*

Si no marcó ninguna casilla en pregunta 1 🡪 *Operacionalización: No discriminación*

**Pregunta 2. A causa de su identidad sexual o sus preferencias sexuales ¿Alguna vez ha sido víctima de violencia y/o agresión? (Opción única)**

Sí 🡪 *Operacionalización: Sí violencia*

No 🡪 *Operacionalización: No violencia*

**Pregunta 3. A causa de su identidad sexual o sus preferencias sexuales ¿Alguna vez ha sido víctima de alguna de las siguientes agresiones? (Opción múltiple)**

Agresión física 🡪 *Operacionalización: Violencia física*

Amenaza 🡪 *Operacionalización: Violencia psicológica*

Agresión verbal 🡪 *Operacionalización: Violencia psicológica*

Chantaje / extorción (amenaza contarle a alguien) 🡪 *Operacionalización: Violencia psicológica*

Violencia sexual 🡪 *Operacionalización: Violencia sexual*

Otra, ¿Cual? ______________________________ 🡪 *Operacionalización:* *(de acuerdo a respuesta brindada)*

**Pregunta 4. ¿En dónde sucedió esta violencia? (Opción múltiple)**

Hogar de crianza 🡪 *Operacionalización: Violencia en entorno cercano/familiar*

Hogar en pareja 🡪 *Operacionalización: Violencia en entorno cercano/familiar*

Trabajo 🡪 *Operacionalización: Violencia en entorno laboral*

Institución educativa 🡪 *Operacionalización: Violencia en instituciones educativas*

Local comercial 🡪 *Operacionalización: Violencia en espacios recreativos/públicos*

Calle o espacio público 🡪 *Operacionalización: Violencia en espacios recreativos/públicos*

Clínica / hospital / Centro de salud/ puesto de salud 🡪 *Operacionalización: Violencia en establecimientos de salud*

Otra, ¿Cuál? _ ______________________ 🡪 *Operacionalización: (de acuerdo a respuesta brindada)*

**Figura 1S. Preguntas empleadas para recopilar información sobre experiencias de discriminación y violencia basada en género. Preguntas han sido adoptadas del cuestionario elaborado por el Centro Latinoamericano de Sexualidad y Derechos Humanos empleado para la encuesta realizada en la ciudad de Bogotá a miembros de la comunidad LGBTQI+ que participaron en la marcha del orgullo de 2007 en dicha ciudad.**
