# Supplementary material for: Depressive symptoms, violence, and discrimination among transgender women during the COVID-19 pandemic: a cross-sectional study
Source: Rev Peru Med Exp Salud Publica. 2025 Sep 12;42(3):281–90. doi: 10.17843/rpmesp.2025.423.14287 (PMC12679979; doi:10.17843/rpmesp.2025.423.14287)
Supplement: Translation

# Translation: Síntomas depresivos, violencia y discriminación en mujeres transgénero durante la pandemia de COVID-19: un estudio transversal

**Authors:** Renato A. Errea, Noris G. Hernandez, Janeth Santa-Cruz, Carmen Contreras, Lourdes Ramos, Diego Rondón, Karen Ramos, Leyla Huerta, Leonid Lecca, Jesús Peinado

**Affiliations:** 1 Socios En Salud Sucursal Perú, Lima, Perú.; 2 School of Social Work, University of South Florida, Tampa, FL, USA.; 3 Escuela Profesional de Tecnología Médica, Universidad Privada San Juan Bautista, Lima, Perú.; 4 Asociación Féminas Perú, Lima, Perú.; 5 Departamento de Salud Global y Medicina Social, Escuela de Medicina de Harvard, Boston, Estado Unidos.; 6 Escuela de Medicina, Facultad de Ciencias de la Salud, Universidad Peruana de Ciencias Aplicadas, Lima, Perú.

**Keywords:** Personas Transfemeninos, Salud Mental, Depresión, Discriminación Basada En Género, Violencia Basada en Género, Perú

## Abstract

**Objetivo.:**

Describir la frecuencia de síntomas depresivos, violencia y discriminación basadas en género en mujeres transgénero (MT) en Lima, durante la pandemia de COVID-19.

**Materiales y métodos.:**

Entre mayo y julio del 2021, la organización no gubernamental Socios En Salud realizó un estudio transversal para evaluar síntomas depresivos, discriminación y violencia basadas en género en MT adultas, identificadas a partir de la base de datos de un estudio previo y mediante referencias de pares. Se describieron las frecuencias y se exploraron las asociaciones entre la sintomatología depresiva y las características de las participantes, incluyendo discriminación y violencia, mediante análisis bivariado. Los síntomas depresivos se evaluaron mediante el cuestionario PHQ-9; la violencia y discriminación, mediante instrumentos adoptados.

**Resultados.:**

Un total de 112 participantes fueron incluidas en el estudio. La mayoría de las MT presentaban síntomas depresivos (81,2%), habían sufrido violencia basada en género (83,8%) y discriminación basada en género (95,5%). La presencia de síntomas depresivos se asoció con experiencias de discriminación en establecimientos de salud (p = 0,046) y con experiencias de violencia (p = 0,042).

**Conclusiones.:**

Se evidenció una alta frecuencia de síntomas depresivos, así como de experiencias de discriminación y violencia basadas en género en MT en Lima, durante la pandemia de COVID-19. Además, se identificaron asociaciones significativas entre dichas experiencias y la presencia de sintomatología depresiva. Estos hallazgos son consistentes con la evidencia sobre el impacto de la pandemia en la salud mental, pero también sugieren una afectación desproporcionada en MT y resaltan la necesidad urgente de abordar los factores que la originan.

## INTRODUCCIÓN

Las mujeres transgénero (MT) presentan una mayor prevalencia de depresión comparada con la población general. Por ejemplo, en Estados Unidos, estudios estimaron una prevalencia de depresión a lo largo de la vida en MT del 62%, mientras que en población general se estimó en 17% ^1^^,^^2^. Asimismo, en comparación con otras minorías sexuales, como los hombres gays y bisexuales, las MT reportan con mayor frecuencia síntomas depresivos ^3^^,^^4^. La depresión deteriora la salud integral y constituye el principal factor de riesgo para el suicidio ^5^^,^^6^. En concordancia con ello, la evidencia también muestra tasas más elevadas de conductas suicidas en personas transgénero en comparación con la población general y otros grupos de género diverso ^7^^,^^8^.

Diversos factores psicosociales contribuyen a la alta prevalencia de depresión en MT. Entre ellos, el estigma, la discriminación y los distintos tipos de violencia (psicológica, física y sexual) se han vinculado al desarrollo de trastornos depresivos en esta población ^4^^,^^9^^-^^13^. Muchas MT viven inmersas en un ciclo de discriminación y violencia que, con frecuencia, se inicia en la niñez o adolescencia, impulsado por la falta de aceptación de identidades de género no normativas por parte de sus familias y de la sociedad ^14^. Acorde a ello, las cifras de discriminación y violencia hacia las MT son elevadas en distintos países del mundo con culturas distintas ^15^^-^^17^.

Estas experiencias de discriminación y violencia favorecen la exclusión social y limitan el acceso a oportunidades educativas y al empleo formal ^14^. En Perú, por ejemplo, cerca del 64% de las MT no ha completado la educación primaria y aproximadamente el 72% ejerce el trabajo sexual como medio de subsistencia ^18^. Esta situación propicia la adquisición de condiciones de salud asociadas tanto a conductas de riesgo como a la pobreza. Por ejemplo, en el país, aproximadamente el 30% de las MT viven con VIH y alrededor del 3,5% presenta tuberculosis activa ^19^^,^^20^.

Por otra parte, la pandemia por COVID-19 generó múltiples estresores como inseguridad, miedo, muerte de personas cercanas, pérdidas socioeconómicas y cambios significativos en la vida cotidiana, los cuales afectaron de manera considerable la salud mental de la población ^21^. Una revisión sistemática estimó que la prevalencia de depresión a nivel global aumentó durante la pandemia, exacerbando la ya elevada carga de esta enfermedad antes del contexto pandémico ^22^. En MT, estudios realizados durante la pandemia, también reportaron una alta frecuencia de síntomas severos de distrés psicológico, con niveles elevados de sintomatología depresiva y superiores a los observados en otras minorías sexo-genéricas ^23^^,^^24^.

Perú fue uno de los países más afectados por la pandemia por COVID-19, con la mayor mortalidad reportada a nivel mundial ^25^. A pesar de ello, se cuenta con escasa evidencia sobre el efecto de ésta sobre la frecuencia de síntomas depresivos en una población altamente vulnerable como la comunidad de MT. Para contribuir a cerrar esta brecha en el conocimiento, el objetivo del presente estudio fue describir la presencia de síntomas depresivos, discriminación y violencia basadas en género, así como explorar los posibles factores asociados a la sintomatología depresiva en MT de Lima, Perú, durante la pandemia de la COVID-19.

**MENSAJE CLAVE**

Motivación para realizar el estudio. Las mujeres transgénero (MT) constituyen una población social e históricamente estigmatizada y marginada, con altas tasas de trastornos de salud mental, incluida la depresión. La pandemia por COVID-19 generó múltiples estresores que deterioraron la salud mental de las personas.

Principales hallazgos. Los hallazgos de este estudio evidenciaron una frecuencia desproporcionadamente elevada de síntomas depresivos en MT en Lima, Perú, en comparación con reportes en población general. Asimismo, se observó altos niveles de discriminación y violencia basadas en género, en concordancia con lo reportado en otros países.

Implicancias. Estos resultados destacan la importancia de abordar estructuralmente el estigma, la discriminación y la violencia contra las MT, incluyendo la implementación efectiva de políticas y leyes antidiscriminatorias.

## MATERIALES Y MÉTODOS

### Tipo y diseño del estudio

La organización no gubernamental Socios En Salud Sucursal Perú (SES) llevó a cabo un estudio transversal con los objetivos de determinar las frecuencias de problemas de salud mental, tuberculosis (TB) y coinfección TB/VIH en MT que residían en Lima. Para la identificación de posibles problemas de salud mental se emplearon cuestionarios, para la detección de TB se empleó radiografía de tórax y pruebas moleculares, y para la determinación del estado serológico con respecto al VIH se realizaron pruebas rápidas o se recopiló la información por autorreporte. Las MT identificadas con alguna de estas condiciones recibieron acompañamiento para recibir el tratamiento respectivo por profesionales de SES o en establecimientos de salud públicos. En este artículo, se reportan los resultados del componente de salud mental, describiendo las frecuencias de síntomas depresivos, de discriminación basada en género y de violencia basada en género en las participantes. Los resultados del componente de TB y de coinfección TB/VIH han sido previamente reportados ^20^.

### Características de la población y selección de la muestra

Las potenciales participantes del estudio tenían 18 años o más, residían en Lima Metropolitana, fueron abordadas entre mayo y julio de 2021 y fueron identificadas por muestreo no probabilístico usando dos fuentes: a) la base de datos de participantes de un estudio transversal realizado por SES en 2020 cuyo objetivo fue describir el acceso a los servicios de atención médica en MT residentes en Lima, o b) referencia directa por pares ^26^. Las MT contactadas y que aceptaron participar del estudio, brindaron su consentimiento para ser evaluadas para uno o más de los tres componentes del estudio (salud mental, TB o coinfección TB/VIH). Las participantes incluidas en este artículo fueron aquellas que aceptaron ser evaluadas por el componente de salud mental.

Durante el periodo de reclutamiento de las participantes, Perú se encontraban en su segunda ola por COVID-19. Para julio de 2021, Perú había reportado más de 2 millones de casos y cerca de 2500 muertes por COVID-19; poco menos de la mitad de los casos y un cuarto de las muertes provenían de Lima ^27^. 

### Medición y categorización de variables

La variable de interés principal del estudio fue la presencia de síntomas depresivos. Para la identificación de estos síntomas, las participantes evaluadas para trastornos de salud mental debían completar primero el Cuestionario de Salud del Paciente-2 (PHQ-2, por sus siglas en inglés). El PHQ-2 es una herramienta de dos preguntas que mide la frecuencia de anhedonia y estado de ánimo decaído durante las dos últimas semanas ^28^. Las participantes que dieron positivo en cualquiera de las dos preguntas del PHQ-2 debían completar el Cuestionario de Salud del Paciente-9 (PHQ-9, por sus siglas en inglés). El cuestionario PHQ-9 es una herramienta de nueve preguntas que evalúa los síntomas depresivos de manera más exhaustiva y evalúa su severidad según el siguiente sistema de puntuación: sin síntomas depresivos (puntuación: 0-4), con síntomas depresivos leves (puntuación: 5-9), con síntomas depresivos moderados (puntuación: 10-14), con síntomas depresivos moderadamente severos (puntuación: 15-19) y con síntomas depresivos severos (puntuación: ≥20) ^29^^,^^30^. La versión en español del PHQ-9 utilizada en el estudio ha recibido una validación por expertos en Perú, asimismo, un estudio local mostró niveles adecuados de confiabilidad y validez para un modelo unidimensional de dicho cuestionario ^31^^,^^32^.

Las variables de interés secundarias fueron la discriminación y la violencia basadas en género. Para ello, se adoptó, con ligeras modificaciones, el contenido de un cuestionario creado y empleado por el Centro Latinoamericano de Sexualidad y Derechos Humanos para una encuesta a miembros de la comunidad LGTBIQ+ en Bogotá, Colombia, en 2007 ^33^. De dicho cuestionario se identificaron las preguntas referentes a discriminación y violencia para recopilar las variables referentes a las experiencias de discriminación basada en género y el entorno donde se experimentó esta discriminación, así como las experiencias de violencia basada en género, el tipo de violencia experimentada y el entorno donde se experimentó esta violencia. El detalle de las preguntas adoptadas y la operacionalización de estas se muestran en el material suplementario.

Finalmente, se recopiló información sobre otras variables de interés del estudio como el estado serológico con respecto al VIH ya sea por autorreporte o por realización de pruebas rápidas de VIH; así como información sociodemográfica correspondiente a la edad, estado civil, nivel educativo y ocupación de las participantes.

### Análisis estadístico

Se realizó un análisis descriptivo reportando la mediana y el rango intercuartílico para la edad (luego de determinar el no cumplimiento de una distribución normal con la prueba de Shapiro-Wilk), y frecuencias absolutas y relativas para el resto de las variables categóricas como las características sociodemográficas de las participantes y las variables de síntomas depresivos, discriminación basada en género y violencia basada en género. Exploratoriamente, se llevó a cabo un análisis bivariado para identificar la asociación entre la presencia de síntomas depresivos con las características demográficas, el estado serológico con respecto al VIH, la discriminación basada en género y la violencia basada en género. Para el análisis bivariado, se dicotomizó el resultado del PHQ-9 en “sin síntomas depresivos” (PHQ < 5) y “con síntomas depresivos” (PHQ ≥ 5); este punto de corte es el indicado por el Ministerio de Salud del Perú para clasificar el resultado del tamizaje de depresión con el cuestionario PHQ-9 como “positivo” o “negativo”, en donde un resultado “positivo” significa un posible diagnóstico de depresión e indica, dentro del flujograma de atención, la necesidad de recibir una consulta de salud mental para confirmación diagnóstica y tratamiento, de corresponder ^34^. Para este análisis bivariado, se empleó la prueba U de Mann Whitney para la comparación con la edad, la prueba exacta de Fisher para variables categóricas cuando el número esperado en más del 20% de las celdas era menor a cinco, y la prueba de chi-cuadrado para variables categóricas que no cumplían criterios para el empleo de la prueba exacta de Fisher. Se consideró significancia estadística para valores de p menores a 0,05. Las participantes con datos faltantes fueron excluidas del análisis. El análisis de datos fue realizado utilizando el programa estadístico STATA v17.0 (Stata Corporation, College Station, Texas, USA).

### Aspectos éticos

El protocolo del estudio principal fue aprobado por el Comité Institucional de Ética en Investigación de Socios En Salud con código #004, cumpliéndose las normas éticas exigidas internacionalmente. Todas las participantes proporcionaron su consentimiento informado por escrito. La gestión y el análisis de datos se realizaron con información no identificable de las participantes.

## RESULTADOS

Un total de 158 participantes dieron su consentimiento para participar en el estudio. De ellas, 142 MT completaron el cuestionario PHQ-2. Según las respuestas del PHQ-2, 114 MT fueron elegibles para completar el cuestionario PHQ-9. Entre las 114 que respondieron el cuestionario PHQ-9, dos MT fueron excluidas debido a datos faltantes sobre violencia y discriminación, lo que resultó en la inclusión de 112 participantes en el análisis (Figura 1).

**Figura 1:** Flujograma de las participantes del estudio.

^a^ PHQ-2, Cuestionario sobre la salud del paciente 2; ^b^ PHQ-9, Cuestionario sobre la salud del paciente 9

La mediana de edad de las 112 participantes del estudio fue de 33,2 años (RIC: 11,1) y el 21,6% no había culminado la educación escolar. Las ocupaciones más frecuentes fueron el trabajo sexual (32,1%) y el estilismo (17,0%); mientras que el 33,9% se encontraba desempleada. Casi un tercio (29,5%) vivía con VIH (Tabla 1).

**Tabla 1:** Características de 112 mujeres transgénero en Lima, Perú, 2021.

Característica		Total n (%)
Edad, mediana (rango intercuartílico)		33,2 (11,1)
Estado civil		
	Soltera	105 (93,8)
	Conviviente	7 (6,2)
Nivel educativo		
	Primaria completa o menos	4 (3,6)
	Secundaria incompleta	19 (17,0)
	Secundaria completa	51 (45,5)
	Superior	38 (33,9)
Ocupación		
	Trabajadora sexual	36 (32,0)
	Estilista	19 (17,0)
	Comerciante	5 (4,5)
	Personal de salud	4 (3,6)
	Cocinera/azafata	3 (2,7)
	Otro	7 (6,3)
	Desempleada	38 (33,9)
Viviendo con VIH		
	Sí	33 (29,5)
	No	79 (70,5)
Ha experimentado alguna vez discriminación basada en género		
	Sí	107 (95,5)
	No	5 (4,5)
Ámbito donde experimentó la discriminación basada en género (n=107)		
	Lugar de trabajo	65 (60,8)
	Entorno familiar/cercano	83 (77,6)
	Establecimientos de salud	50 (46,7)
	Instalaciones religiosas	32 (29,9)
	Instituciones educativas	54 (50,5)
	Instalaciones judiciales/policiales	84 (78,5)
	Espacios recreativos	51 (47,7)
Ha experimentado alguna vez violencia basada en género		
	Sí	94 (83,9)
	No	18 (16,1)
Tipo de violencia basada en género experimentada (n=94)		
	Física	46 (48,9)
	Psicológica	94 (100,0)
	Sexual	22 (23,4)
Ámbito donde experimentó la violencia basada en género (n=94)		
	Entorno familiar/cercano	16 (17,0)
	Lugar de trabajo	5 (5,3)
	Instituciones educativas	4 (4,3)
	Espacios recreativos	54 (57,5)
	Establecimientos de salud	0 (0,0)
	No especifica	15 (16,0)
Síntomas depresivos		
	Sin síntomas	21 (18,8)
	Síntomas leves	54 (48,2)
	Síntomas moderados	23 (20,5)
	Síntomas moderadamente severos	11 (9,8)
	Síntomas severos	3 (2,7)

a Mediana (rango intercuartílico). La variable “edad” no presentó una distribución normal (prueba de Shapiro-Wilk: W = 0.954, valor de p < 0,001)

En cuanto a las experiencias de discriminación debido a su identidad de género, el 95,5% informó haber sufrido discriminación, entre ellos, los entornos más frecuentes fueron en las instalaciones judiciales/policiales (78,5%) y en el entorno familiar/cercano (77,6%). El 83,9% informó experiencias de violencia debido a su identidad de género, siendo la violencia psicológica la más frecuente (100%), y el entorno más común en los espacios recreativos (57,5%), seguido del entorno familiar/cercano (17,0%). La mayoría de las participantes presentaban algún grado de síntomas depresivos (81,2%), con predominio de síntomas depresivos leves (48,2%) y síntomas depresivos moderados (20,5%).

El análisis exploratorio mostró una asociación estadísticamente significativa entre aquellas participantes que experimentaron discriminación basada en género en establecimientos de salud (p = 0,046) y entre aquellas que experimentaron violencia basada en género (p = 0,042) (Tabla 2).

**Tabla 2:** Análisis bivariado de los síntomas depresivos y otras características de 112 mujeres transgénero en Lima, Perú, 2021.

Característica			Sín síntomas depresivos (N=21) n (%)	Con síntomas depresivos (N=91) n (%)	Valor de p
Edad, mediana, (rango intercuartílico)			35,0 (8,8)	32,8 (11,7)	0,751^a^
Estado civil					
	Soltera		21 (20,0)	84 (80,0)	0,344^b^
	Conviviente		0 (0,0)	7 (100,0)	
Nivel educativo					
	Educación secundaria o menos		12 (16,2)	62 (83,8)	0,338^c^
	Educación superior		9 (23,7)	29 (76,3)	
Ocupación					
	Trabajadora sexual		6 (16,7)	30 (83,3)	0,448^b^
	Estilista		2 (10,5)	17 (89,5)	
	Otros		6 (31,6)	13 (68,4)	
	Desempleada		7 (18,4)	31 (81,6)	
Persona viviendo con VIH					
	Sí		7 (21,2)	26 (78,8)	0,666^c^
	No		14 (17,7)	65 (82,3)	
Experiencia de discriminación basada en género					
	Sí		20 (18,7)	87 (81,3)	1,00^b^
	No		1 (20,0)	4 (80,0)	
Ámbito donde experimentó la discriminación basada en género (n=107)					
	Lugar de trabajo				
		Sí	11 (16.9)	54 (83,1)	0.559^c^
		No	9 (21,4)	33 (78,6)	
	Entorno familiar/cercano				
		Sí	14 (16,9)	69 (83,1)	0,382^b^
		No	6 (25,0)	18 (75,0)	
	Establecimientos de salud				
		Sí	5 (10,0)	45 (90,0)	0,031^c^
		No	15 (26,3)	42 (73,7)	
	Instalaciones religiosas				
		Sí	5 (15,6)	27 (84,4)	0,595^c^
		No	15 (20,0)	60 (80,0)	
	Instituciones educativas				
		Sí	8 (14,8)	46 (85,2)	0,299^c^
		No	12 (22,6)	41 (77,4)	
	Instalaciones judiciales/policiales				
		Sí	16 (19,0)	68 (81,0)	1,000^b^
		No	4 (17,4)	19 (82,6)	
	Espacios recreativos				
		Sí	7 (13,7)	44 (86,3)	0,209^c^
		No	13 (23,2)	43 (76,8)	
Experiencia de violencia basada en género					
	Sí		14 (14,9)	80 (85,1)	0,042^c^
	No		7 (38,9)	11 (61,1)	
Tipo de violencia basada en género experimentada (n=94)					
	Física				
		Sí	8 (17,4)	38 (82,6)	0,506^c^
		No	6 (12,5)	42 (87,5)	
	Psicológica				
		Sí	14 (14,9)	80 (85,1)	N/A^d^
		No	0 (0,0)	0 (0,0)	
	Sexual				
		Sí	6 (27,3)	16 (72,7)	0,086^b^
		No	8 (11,1)	64 (88,9)	
Ámbito donde experimentó la violencia basada en género (n=94)					
	Entorno familiar/cercano				
		Sí	2 (12,5)	14 (87,5)	1,000^b^
		No	12 (15,4)	66 (84,6)	
	Lugar de trabajo				
		Sí	1 (20,0)	4 (80,0)	0,562^b^
		No	13 (14,6)	76 (85,4)	
	Instituciones educativas				
		Sí	0 (0,0)	4 (100,0)	1,000^b^
		No	14 (15,6)	76 (84,4)	
	Espacios recreativos				
		Sí	6 (11,1)	48 (88,9)	0,231^c^
		No	8 (20,0)	32 (80,0)	

a El valor de p fue calculado con la prueba U de Mann-Whtiney.

b El valor de p fue calculado con la prueba exacta de Fisher.

c El valor de p fue calculado con la prueba de chi-cuadrado.

d Prueba estadística no aplicable: fila con frecuencia 0.

## DISCUSIÓN

Se encontró una alta frecuencia (81,2%) de síntomas depresivos en MT en Lima, Perú, durante la pandemia de COVID-19. Un estudio local en MT de Lima realizado antes de la pandemia reportó una menor frecuencia de síntomas depresivos (PHQ ≥ 5) en MT respecto al presente estudio (60% vs 81.2%, respectivamente), aunque dicho estudio contó con un tamaño de muestra pequeño ^35^. Asimismo, la frecuencia de síntomas depresivos de moderados a más (PHQ ≥ 10) fue mayor que la prevalencia reportada por un estudio local realizado durante la pandemia en población general (33,0% vs 19,7%, respectivamente) ^36^. Estos hallazgos sugieren que las condiciones estresoras de la pandemia han afectado desproporcionadamente a la salud mental de las MT en comparación con la población general, generando elevada sintomatología depresiva.

La violencia (83,9%) y la discriminación basadas en género (95,5%) presentaron cifras elevadas en la población estudiada. En cuanto a violencia, se reportaron altas frecuencias de violencia física (48,9%) y psicológica (100%), mientras que la violencia sexual fue menor (23,4%). A excepción de la violencia sexual, estos hallazgos son comparables a los reportados en un estudio realizado en Brasil, que encontró prevalencias elevadas de violencia sexual (47,5%), física (54,2%), psicológica (85,8%) y de discriminación (91,6%) ^37^. Por otra parte, un estudio en Irán reportó una frecuencia similar de violencia psicológica (92%), con mayor frecuencia de violencia física (70,9%) y sexual (63%) ^15^. A pesar de algunas variaciones, estos hallazgos reflejan la magnitud de la violencia y discriminación que enfrentan las mujeres transgénero en distintos contextos socioculturales.

En el presente estudio, el análisis bivariado exploratorio identificó una asociación significativa entre los síntomas depresivos durante la pandemia y la violencia basada en género. Este hallazgo es consistente con la literatura que documenta asociaciones positivas entre la violencia (psicológica, física o sexual) y la depresión mayor en contextos no pandémicos ^9^^,^^10^. Asimismo, se observó una asociación significativa entre la presencia de síntomas depresivos y experiencias de discriminación basada en género en entornos de atención médica. Este resultado reviste particular importancia en el contexto peruano, donde un estudio realizado en cinco regiones reportó que cerca del 30% de MT habían sufrido discriminación en algún establecimiento de salud en los últimos doce meses ^38^; estas mismas experiencias de discriminación pueden generar negación para la búsqueda de servicios de salud mental en los establecimientos. La salud mental de las MT puede verse favorecida mediante la implementación de servicios de salud inclusivos ^(^^39^. No obstante, para abordar de forma integral la violencia y la discriminación, es fundamental impulsar marcos legales antidiscriminatorios efectivos ^40^, y desarrollar intervenciones dirigidas hacia los mismos perpetradores ^(^^41^.

La frecuencia de síntomas depresivos entre las MT viviendo con VIH y aquellas que no fue similar (78,8% y 82,8%, respectivamente). De manera consistente, un estudio transversal en Brasil que utilizó el cuestionario PHQ-9 tampoco encontró diferencias en la presencia de síntomas depresivos según el estado serológico con respecto al VIH en MT ^9^. Sin embargo, la literatura señala que la depresión constituye un factor predisponente para involucrarse en prácticas sexuales de riesgo en MT ^42^, incluso mayor al comparado con hombres que tienen sexo con hombres ^43^. Considerando que uno de los principales puntos de contacto de las MT con el sistema de salud son los servicios de VIH y de infecciones de transmisión sexual (ITS), ya sea para pruebas de detección o para el acceso a tratamiento, se presenta una oportunidad clave para integrar la atención de salud mental dentro de estos servicios ^44^. Esta integración cobra mayor relevancia ante la escasez de personal especializado en salud mental en muchos países de ingresos bajos y medianos, como Perú. Con relación a ello, las intervenciones de baja intensidad para el abordaje de la depresión (es decir, intervenciones implementadas por personal no especializado en salud mental pero entrenado), han demostrado ser efectivas en servicios de VIH/ITS en estos contextos ^45^. Implementar estas estrategias en el país podría facilitar el acceso a servicios de salud mental para las MT.

En contextos de pandemia, caracterizados por severas restricciones de movilización como las cuarentenas, las intervenciones basadas en salud digital pueden disminuir el estrés psicológico y la sintomatología depresiva. En línea con ello, un metaanálisis reveló que intervenciones basadas en terapia cognitivo-conductual impartidas a través de telemedicina resultaron efectivas para disminuir los síntomas de depresión en población general durante la pandemia por COVID-19 ^46^. Estas intervenciones variaban en su formato: algunas eran conducidas por personal de salud, otras eran autoguiadas; algunas se ofrecían en formatos abreviados y otras en formatos más extensos. En contraste, la evidencia sobre el uso de la salud mental digital en minorías sexuales y de género durante este periodo es escasa. Un estudio cuasiexperimental implementado durante la pandemia por COVID-19 en una muestra de personas pertenecientes a minorías sexuales y de género, incluyendo personas transgénero, demostró que una intervención breve, de tres sesiones e impartida por pares, logró reducir significativamente los síntomas depresivos ^47^. Estos hallazgos evidencian el potencial de las intervenciones digitales en salud mental durante escenarios pandémicos, pero también subrayan la necesidad urgente de generar evidencia específica y adaptada para minorías sexuales y de género.

Este estudio presenta limitaciones. En primer lugar, se excluyó del análisis a las participantes que puntuaron negativo al cuestionario PHQ-2, lo que podría haber sobreestimado el porcentaje de personas con síntomas depresivos; esta exclusión se debió a que, en dichas participantes, no se recolectaron datos sobre las demás variables relevantes del estudio. En segundo lugar, si bien los síntomas depresivos se midieron mediante el cuestionario PHQ-9, una herramienta validada en Perú y ampliamente recomendada para el tamizaje de depresión en distintos contextos ^31^^,^^32^^,^^48^, ésta no ha sido validada ni adaptada para su uso en población transgénero en Perú, lo que podría haber afectado la confiabilidad y validez de los resultados. De igual manera, los instrumentos utilizados para recolectar información sobre discriminación y violencia basada en género no fueron validados ni adaptados al contexto de la población transgénero; lo que también podría haber influido en la confiabilidad y validez de estas mediciones. Por otra parte, el muestro no probabilístico empleado podría haber introducido sesgos de selección. Además, el muestreo por referencia de pares podría haber introducido sesgos de selección adicionales, al favorecer la inclusión de participantes con características sociales o conductuales similares. Ambos factores limitan la validez externa de los resultados. Finalmente, el limitado número de participantes restringió la potencia estadística del estudio y no permitió realizar análisis ajustados. No obstante, es importante considerar que las MT constituyen una población de difícil acceso y que otros estudios en esta población enfrentan desafíos de muestreo similares.

En conclusión, se evidenció una frecuencia elevada de síntomas depresivos, discriminación y violencia basadas en género en MT en Lima, Perú, durante la pandemia por COVID-19; así como asociaciones de las experiencias de discriminación y violencia con la presencia de sintomatología depresiva. Los hallazgos sugieren un impacto desproporcionado de la pandemia por COVID-19 en la salud mental de las MT, lo que subraya la importancia de atender los factores estructurales y sociales que lo generan.
